# Emphysematous cystitis with clinical subcutaneous emphysema

**DOI:** 10.1186/1865-1380-4-26

**Published:** 2011-06-13

**Authors:** Ahmed-Ramadan Sadek, Helen Blake, Anand Mehta

**Affiliations:** 1 Care of the Elderly Medicine Department Mayday University Hospital, 530 London Road Croydon, CR8 2YL, UK

## Abstract

Emphysematous cystitis (EC) is the presence of intramural gas, with or without luminal gas, within the bladder as a result of a primary infection of the lower urinary tract with a gas-producing organism. It is a well-recognised complication of urinary tract infections involving *Escherichia coli *in diabetic patients. Clinical subcutaneous emphysema is a rare complication of EC that appears to have poor prognosis. Only careful clinical judgement, and a high degree of suspicion, will lead to its early diagnosis and treatment. Here, we report a case of subcutaneous emphysema due to EC based on a clinical diagnosis confirmed using computed tomography (CT).

## Introduction

Emphysematous cystitis (EC) is the presence of intramural gas, with or without luminal gas, within the bladder as a result of a primary infection of the lower urinary tract with a gas-producing organism. The spectrum of clinical presentation of EC is non-specific and can range from minimally symptomatic urinary tract infection (UTI) to a scenario of peritonitis and septic shock [1]. Here, we report a case of subcutaneous emphysema due to EC based on a clinical diagnosis confirmed using computed tomography (CT).

### Case report

An 81-year-old lady with poorly controlled non-insulin dependent diabetes presented to our accident and emergency department with increased frailty and confusion following review by her GP. She had been discharged 10 days earlier following treatment for a lower respiratory tract infection. In view of her deterioration, her GP was concerned that her decline may have been attributable to an occult infection or neoplasm. She was observed to have bilateral pitting oedema. On assessment in hospital, the patient was described as being "cushingoid" in appearance, and there was generalised crepitus on abdominal palpation to the infra-mammary region. The remainder of the clinical examination was unremarkable.

**Figure 1:**
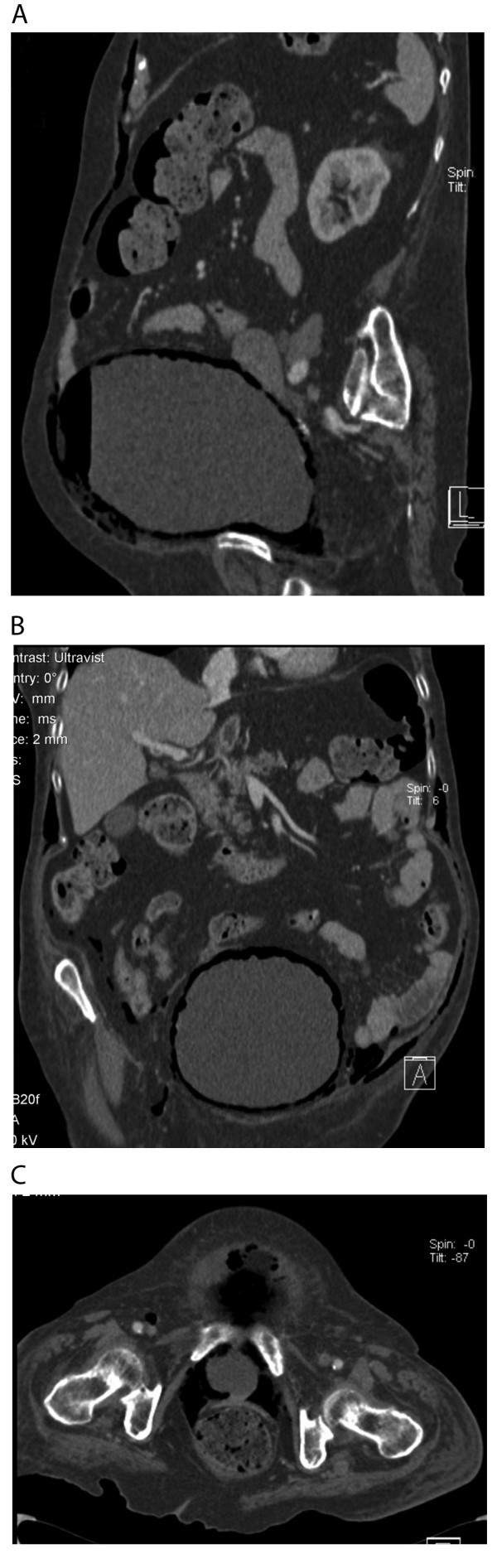
**Radiographic imaging of subcutaneous emphysema secondary to emphysematous cystitis**. **a **CT sagittal section showing gas within the anterior abdominal wall and within the bladder lumen and wall. **b **CT coronal section showing gas within abdominal wall outside the muscle layer, intra-abdominal but retroperitoneal, and within the bladder wall. **c **CT axial section showing gas within the bladder base and femoral canal.

A CT scan was requested to define the aetiology of the clinical findings. The CT scan demonstrated a hugely distended bladder with an air fluid level and intramural gas (Figure 1a). In addition, gas was observed in several other bodily compartments; in the anterior abdominal wall (Figure 1b), intra-abdominal but extra-peritoneal (no free intraperitoneal gas), infra-peritoneal (within the pelvis), and the femoral canal (Figure 1c).

A urine culture grew *Escherichia coli *(*E. coli*), and on the basis of sensitivities the patient was started on a course of gentamicin and ciprofloxacin. Over the ensuing 2 days she continued to deteriorate, and the white cell count remained raised (WCC 13.5 × 10^9^/l). On the advice of the on-call surgical team and the consultant microbiologist, the antibiotic regimen was changed to intravenous ceftazidine and metronidazole with oral cefalexin. Although it was a possibility, there was no evidence to suggest the presence of an enterovesical fistula, and her physical frailty precluded invasive investigation.

Three days later she developed right-sided pneumonia, for which she was started on a course of oral clarithromycin. In consultation with family it was decided to treat the patient conservatively. All intravenous antibiotics were stopped, and the patient passed away 5 days later (a total of 21 days after her most recent admission).

Prior to this admission and above diagnoses, the patient's other significant medical history included essential hypertension, diverticulosis, leg cramps, orthostatic pedal oedema, previous mastectomy of the left breast for invasive ductal carcinoma, right delta shoulder joint replacement following a rotator cuff tear and hiatus hernia. She was on once daily glimerpiride 4 mg, simvastatin 40 mg, quinine sulphate 300 mg, amiloride 50 mg and frusemide 40 mg; twice daily loperamide 2 mg, metformin 1 g and ranitidine 150 mg; and three times daily metoclopramide 10 mg and temezepam 10 mg pro re nata.

## Discussion

While there have been over 130 cases of EC documented in the literature [1], there has only ever been, up to now, one published case report of EC presenting with subcutaneous emphysema [2]. Using plain radiographs and a cystogram, this earlier case report from 1978 described luminal and possible intramural gas within the bladder and the presence of subcutaneous emphysema in the absence of a possible vesicoenteric fistula. We report the first case of subcutaneous emphysema due to EC based on a clinical diagnosis confirmed using CT.

Emphysematous cystitis, per se, was first described in 1671 when a patient was said to have passed wind (i.e., intraluminal gas) through his urethra [3]. A little later in the 1800s intramural gas was discovered on autopsy [4]. And in 1961 a review [5] of multiple cases concluded that the two conditions (i.e. intraluminal and intramural gas) were manifestations of a single disease. The clinical presentation of EC is variable; approximately 53% of cases present with classical symptoms of urinary tract infection [6], whilst others may present with an acute abdomen [7]. Up to 7% of cases are asymptomatic and are diagnosed on the basis of an incidental finding on abdominal/pelvic imaging [6].

In spite of the variation in clinical presentation of EC, type 2 diabetes mellitus has been shown to be present in 2/3 of all cases, and of these 64% were women [1,6]. These figures may well be even higher as a 1/3 of all cases of diabetes mellitus are undiagnosed [8]. Moreover, with the predicted doubling of the prevalence of type 2 diabetes from 1995 to 2025 [9], clinicians need to be aware of its role as possible pathological basis for a complicated or un-resolving UTI.

It is postulated that the presence of gas-producing organisms in conjunction with high glucose or albumin concentrations (both bacterial substrates) favours the development of emphysematous infections within the urinary tract [10]. In 90% of cases of EC a urinary tract pathogen was isolated [6], and *E. coli *was the most prevalent pathogen (57%). It may very well be that the remaining 10% of cases where a urinary tract pathogen was not isolated may be attributable to detection failure. Indeed, it was only on the second urine culture that *E. coli *was isolated in our case.

It is tempting to speculate the anatomical route taken by the perivesicular gas to produce the radiological imaging observed in this case study. It is probable that the carbon dioxide bubbles produced as a result of urinary glucose fermentation collect and pass into the submucosa of the bladder and out into the infra-peritoneal space around the bladder base. Once here these bubbles may move across and diffuse into and through abdominal musculature. Similarly they may move down into the ischiorectal fossa or up through the retroperitoneal paravertebral tissues into the posterior mediastinum [2].

The management of EC has remained unchanged over the last 30 years [2], with broad-cover intravenous antibiotics being used until urinary pathogen sensitivities are known. Concurrently, the bladder should be drained and blood glucose levels should be controlled. Between 10-20% of documented patients with EC underwent surgical debridement [1,6]. As carbon dioxide is absorbed readily in human tissue, eventual resolution should occur following antibiotic elimination of the infecting pathogen; hence, the precise role of surgical intervention is not clear. By itself, EC usually runs a benign course with an overall death rate of 7% [1]; this however rises to almost 50% when perivesicular gas migrates up the urinary tract or when gas-producing organisms infect the kidneys [11,12]. It is likely that patients with EC with extensive emphysematous changes will have a worse prognosis because of the greater distribution of perivesicular gas.

## Conclusion

In conclusion, clinical subcutaneous emphysema is a rare complication of EC that appears to have poor prognosis. Only careful clinical judgement, and a high degree of suspicion, will lead to its early diagnosis and treatment. Its true incidence and specific management can only be determined by greater awareness of its presence as a rare but important complication of urinary tract infection, especially in patients with poorly controlled diabetes.

The use of CT has identified that the term subcutaneous emphysema does not convey the extent of perivesicular emphysema, and we suggest the term "multi-compartment emphysema" more accurately describes our findings.

## Key points

1. Emphysematous cystitis is a well-recognised complication of urinary tract infections involving *E. coli *in diabetic patients.

2. Computed tomography is ideal in defining vesicular and perivesicular emphysema.

3. Prompt aggressive management using intravenous antibiotics is the most appropriate way to treat EC.

## Abbreviations

EC: emphysematous cystitis; UTI: urinary tract infection; CT: computerised tomography.

## Competing interests

The authors declare that they have no competing interests.

## Authors' contributions

ARS wrote the first draft of the paper and contributed to all drafts. HB reviewed and commented on all the drafts of the paper and radiographic images. AM contributed to all drafts of the paper.

## Consent

Consent was obtained from the patient for the publication of this case report and the accompanying images.
